# Influence of Solvent Challenge on the Hardness and Toughness of Viscosity-Modified Composites

**DOI:** 10.3390/bioengineering13070726

**Published:** 2026-06-24

**Authors:** Abdulrahman Alshabib, Hamad Algamaiah, Silvia Rojas-Rueda, Carlos A. Jurado, Abdullah Almansour, Saad AlOtaibi

**Affiliations:** 1 Department of Restorative Dental Science, College of Dentistry, King Saud University, Riyadh 11545, Saudi Arabia; 2 School of Dental Medicine, Ponce Health Sciences University, Ponce, PR 00732, USA; 3 College of Dentistry, Prince Sattam Bin Abdulaziz University, Al-Kharj 16245, Saudi Arabia

**Keywords:** composite resins, hardness, fracture toughness, aging, dental materials, polymers

## Abstract

Background: This study assessed the effect of solvent aging on Vickers microhardness (VHN) and fracture toughness (K_IC_) of four resin composites: two high-strength flowable materials indicated for all cavity classes (GUF and CBF), a packable nanohybrid composite (XTE), and its flowable counterpart (XTEF). Methods: Disk specimens (*n* = 6 per group) were photocured and stored at 37 °C in distilled water or in a 75%/25% ethanol/water solution for 1 or 30 days. Vickers microhardness was recorded with a 300 g load applied for 15 s. Single-edge-notched beam (SENB) specimens (32 × 6 × 3 mm; *n* = 6 per group) were loaded in three-point bending for K_IC_ determination after 1 and 30 days of storage in water or ethanol/water. Data were analyzed by three-way ANOVA followed by Tukey’s post hoc test (α = 0.05). Results: At 1 day, the four composites separated into distinct VHN groups in the order XTE > GUF > CBF > XTEF (*p* ≤ 0.05). After 30 d, VHN decreased in all materials, with larger reductions in ethanol/water (17–30% relative to the 1 d water value) than in water alone (5–11%). At 1 day, K_IC_ values for XTE, GUF and CBF formed a single statistical group, all significantly higher than XTEF (*p* ≤ 0.05). Thirty-day water storage did not affect K_IC_ for any material (*p* > 0.05), whereas ethanol/water storage reduced K_IC_ by 21–26% in all four composites and produced four distinct material groups (*p* ≤ 0.05). Conclusions: The high-strength flowable composites showed greater hardness and toughness than a conventional flowable composite but did not match the mechanical performance of the highly filled packable composite. Ethanol/water aging markedly softened the composite surfaces and reduced fracture toughness, whereas prolonged water storage had a smaller effect on hardness and no measurable effect on K_IC_.

## 1. Introduction

Flowable resin composites were introduced as low-viscosity restoratives to improve cavity-wall adaptation and simplify placement. Relative to conventional packable composites, traditional flowables contain approximately 20 wt.% less filler, a compositional trade-off that improves handling but reduces surface hardness, elastic modulus, wear resistance, and fracture resistance [1]. Their clinical use has therefore been restricted to low-stress indications such as liners, small restorations, and areas in which enhanced marginal adaptation is desired. More recent formulations, variously marketed as “high-strength” or “universal” flowables, aim to preserve the handling advantages of flowable materials while raising filler loading and modifying the matrix chemistry to approach the mechanical behavior of packable composites, including reduced polymerization shrinkage stress, improved wear resistance, and higher flexural strength [2,3,4,5,6,7,8].

Published data suggest that some high-strength flowables now approach the flexural strength of conventional packable composites [3,9]. A 36-month randomized clinical trial of a highly filled flowable in posterior restorations reported clinical performance comparable to a conventional paste composite [10]. These findings raise the question of whether the performance gap between flowable and packable composites has genuinely narrowed or whether it re-opens under conditions that stress the resin matrix.

Two mechanical descriptors are central to answering this question. Surface hardness reflects resistance to plastic deformation and correlates with wear behavior and resistance to abrasion. In resin composites, hardness is governed by the degree of conversion and by filler content, size, and coupling chemistry; highly filled, well-polymerized composites exhibit the highest Vickers values [1,11]. Flowable composites, with their larger resin fraction, typically show lower hardness than densely filled packable materials [11,12]. Fracture toughness (K_IC_) describes resistance to unstable crack propagation and is the most relevant single parameter for predicting mechanical survival in load-bearing restorations [13,14]. Conventional hybrid composites have K_IC_ values in the range of 1–2 MPa·m^0.5^, with flowables typically at the lower end of this interval [1]. Attempts to raise K_IC_ in flowable materials have focused on filler architecture (nanoclusters, prepolymerized fillers, short glass fibers) and on modifications to the monomer system [1,15,16].

Aging plays a decisive role in how these mechanical indices evolve in service. In the oral cavity, resin composites are continuously exposed to saliva, to food-simulating liquids, and, intermittently, to organic solvents such as the ethanol present in alcoholic beverages and mouthrinses. Water sorption plasticizes the polymer network, promotes hydrolytic cleavage of ester bonds in the dimethacrylate matrix, and attacks the silane layer at the filler–matrix interface [17,18]. Ethanol penetrates the network more rapidly than water; swelling the matrix relaxes internal stresses at the interphase, accelerates leaching of unreacted monomer, and provides the kinetic conditions for filler debonding and micro-crack initiation [17,19]. The 75% ethanol/water mixture specified as a food-simulating liquid in ISO 10993-12 is a widely accepted accelerated-aging medium in dental materials research [17,20,21]. Hardness losses of approximately 20% after short-term ethanol storage are commonly reported, whereas pure water produces milder changes over comparable periods [1,17,19]. K_IC_ appears less sensitive to short-term water storage [1,22], but sustained exposure to more aggressive media can compromise crack resistance by degrading both the matrix and the filler–matrix interphase [17,18,22].

Recent in vitro studies report that injectable and high viscosity bulk-fill flowables can approach conventional packable composites in flexural strength and core build-up performance, yet their behavior under prolonged solvent challenge has not been systematically benchmarked against a packable control [23,24]. Information on how the current generation of “all-class” flowable composites compares with conventional materials under prolonged solvent aging remains limited. Addressing this gap is clinically meaningful because it directly relates to the anticipated longevity of restorations placed with these materials. Although the general influence of filler loading and solvent exposure on composite mechanics is well established, these newly introduced high-strength flowables are being marketed for load-bearing posterior use and have not previously been characterized under standardized solvent aging against a packable benchmark; the present study addresses that specific gap and, in doing so, tests whether bulk filler content or the resin matrix/interface governs solvent-aged crack resistance. The aim of the present study was therefore to quantify the effect of solvent aging on the surface hardness and fracture toughness of two high-strength flowable resin composites (GUF and CBF) and to benchmark their performance against a highly filled nanohybrid packable composite (XTE) and its flowable counterpart (XTEF). The null hypotheses tested were that (i) the four composites do not differ in hardness or fracture toughness, and (ii) neither storage medium nor storage time modifies these properties.

## 2. Materials and Methods

**Figure A1:**
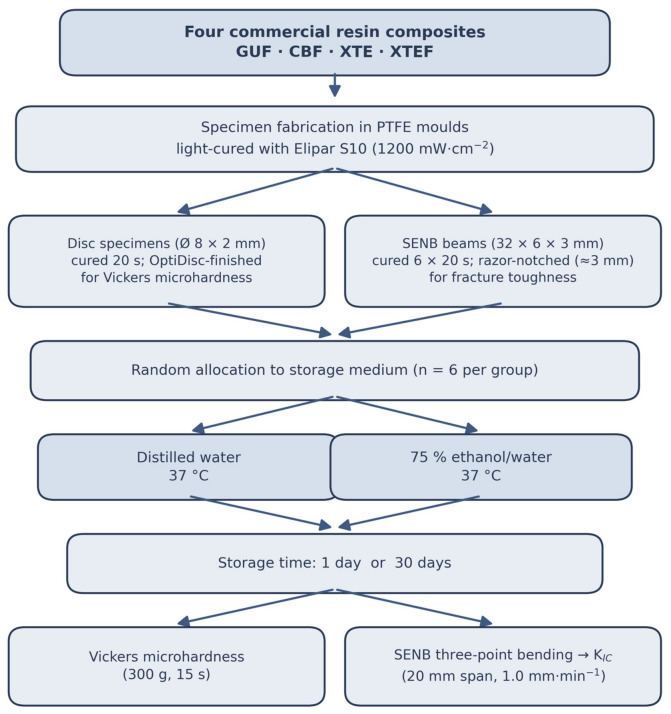
Schematic of the study design and experimental workflow, from the four commercial resin composites through specimen fabrication, random allocation to storage medium, solvent aging, mechanical testing, and statistical analysis.

Four light-cured resin composites were evaluated (Table 1): G-ænial Universal Flo (GUF), a nanohybrid “universal” flowable composite (GC Corp., Tokyo, Japan); Charisma Bulk Flow ONE (CBF), a bulk-fill one-shade flowable composite (Kulzer GmbH, Hanau, Germany); Filtek Z350 XT (XTE), a nanohybrid packable composite (3M ESPE, St. Paul, MN, USA), used here as the positive control; and Filtek Z350 XT Flowable (XTEF), the flowable counterpart of XTE, used as a reference for conventional flowable behavior. All materials were used in shade A2 (or the single universal shade, for CBF) (Table 1). The overall study design and experimental workflow are summarized in Figure A1 (Appendix A).

### 2.1. Surface Hardness

Disk specimens (8 mm diameter × 2 mm thickness) were prepared in PTFE molds. A Mylar strip lined the base of the mold; after the mold was filled, a second Mylar strip and two 1 mm glass slides were applied and clamped to extrude excess material and ensure uniform thickness. Photo-polymerization was carried out through the upper slide for 20 s with an LED curing unit (Elipar S10, 3M ESPE, Seefeld, Germany) delivering an irradiance of 1200 mW·cm^−2^, verified before each cycle with a MARC™ Resin Calibrator (Blue Light Analytics, Halifax, NS, Canada).

After demolding, both surfaces of each specimen were finished with an OptiDisc abrasive set (coarse→medium→fine; Kerr Hawe SA, Bioggio, Switzerland) mounted in a contra-angle handpiece operated at a rotational speed of 15,000 rpm to remove flash and standardize surface roughness.

**Figure 1:**
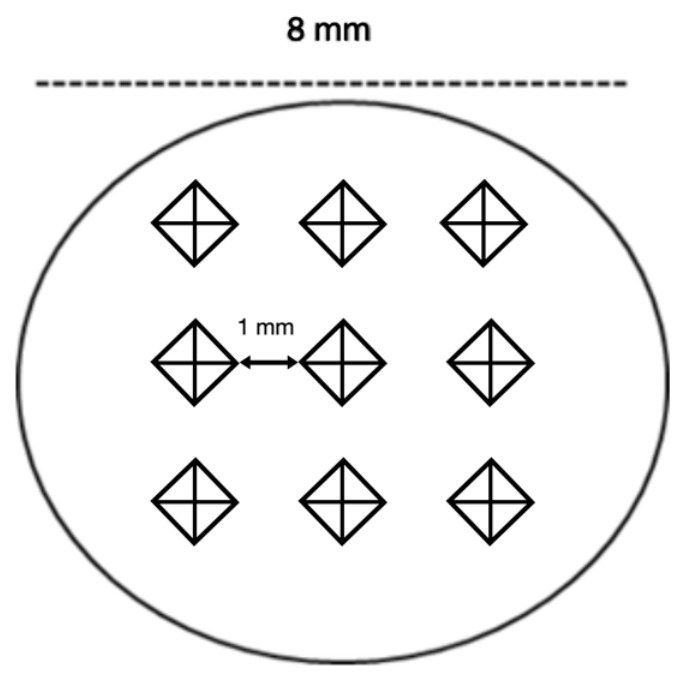
Schematic illustration of the 3 × 3 Vickers indentation pattern, showing the nine indentations per specimen separated by approximately 1 mm.

For each composite, specimens were allocated randomly to one of two storage media (*n* = 6 per group): distilled water or a 75 vol.% ethanol/water solution. Vickers microhardness (VHN) was recorded after 1 d and 30 d of storage at 37 ± 1 °C in the dark. Indentations were produced with a microindenter (Nova 130/240 series, INNOVATEST Europe BV, Borgharenweg, The Netherlands) under a 300 g load applied for 15 s at 23 ± 1 °C. Nine indents were placed on each specimen in a 3 × 3 pattern, each separated by at least 1 mm and positioned ≥1 mm from the specimen edge (Figure 1); the mean of the nine indents was taken as the VHN of that specimen, in accordance with ASTM E384 [25].

### 2.2. Fracture Toughness

Twelve Single-edge-notched beam (SENB) specimens (32 × 6 × 3 mm; *n* = 6 per group) were prepared in PTFE-lined brass molds. Uncured composite was packed into the mold, overfilled, and covered with Mylar film and glass slides. Photo-polymerization was performed using the curing unit described in Section 2.1 for a cumulative 120 s, delivered as six overlapping 20 s exposures to ensure uniform conversion along the beam length. A sharpened razor blade was used to introduce a ≈ 3 mm notch at mid-span.

Residual flash was removed with 320-grit silicon carbide paper, after which the beams were stored in distilled water at 37 °C for 24 h. Initial notch length (*a*_0_) was measured with a stereomicroscope (EMZ 5, Meiji Techno, Saitama, Japan) at 1.5× magnification and recorded to 0.1 mm. Specimen depth (*B*) and width (*W*) were measured at three equidistant locations with a digital caliper (resolution ± 0.01 mm).

**Figure 2:**
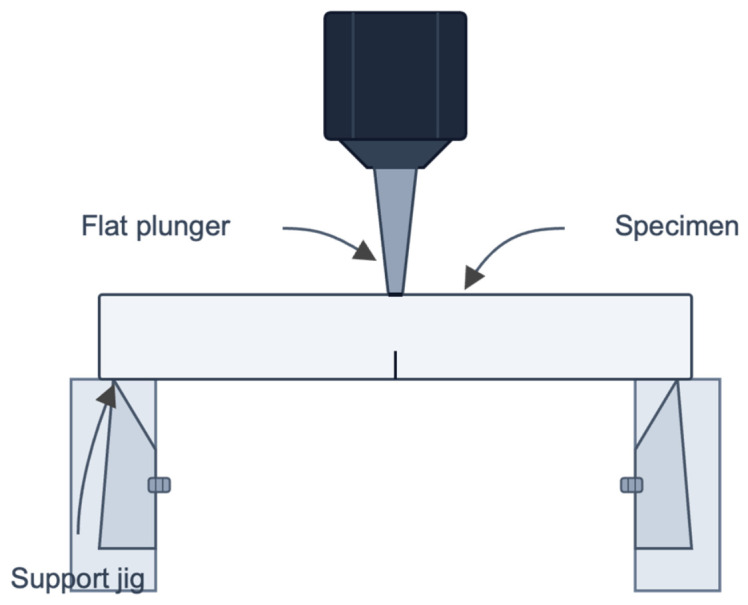
Schematic illustration of the SENB specimen mounted on the three-point bending jig of the Instron universal testing machine (flat plunger: 8 mm length × 1.2 mm width).

Fracture toughness (K_IC_) was determined by three-point bending on a universal testing machine (Instron model 5965, Instron Corp., Canton, OH, USA) with a 20 mm support span (Figure 2). Tests were run at 23 ± 1 °C and at a crosshead speed of 1.0 mm·min^−1^ until catastrophic failure. The critical load (*P*_c_) was used to calculate K_IC_ according to the linear elastic fracture mechanics expression for SENB specimens, as specified in ASTM D5045 and ISO 13586 for polymeric materials [26,27]:

(1) $$K_{IC}=[\frac{PL}{{BW}^{1.5}}]Y$$

P = Load at fractureB = thickness of the specimenL = distance between the supportsY = calibration function for given geometryW = width of the specimena = notch lengthY = [2.9 (*a/w*)^1/2^ − 4.6 (*a/w*)^3/2^ + 21.8 (*a/w*)^5/2^ − 37.6 (*a/w*)^7/2^ + 38.7 (*a/w*)^9/2^]Equation (1): Fracture toughness equation

### 2.3. Statistical Analysis

Data were analyzed in SPSS 27.0 (IBM Corp., Armonk, NY, USA). Homogeneity of variance was verified with Levene’s test and the assumption of equal variance was satisfied. A three-way ANOVA (factors: material, storage medium, storage time) followed by Tukey’s HSD post hoc test was used to examine the main effects and the two- and three-way interactions on Vickers microhardness and on fracture toughness. The significance level was set at α = 0.05; effects with *p* ≤ 0.05 are reported as significant together with the associated *F* statistic and degrees of freedom.

## 3. Results

**Figure 3:**
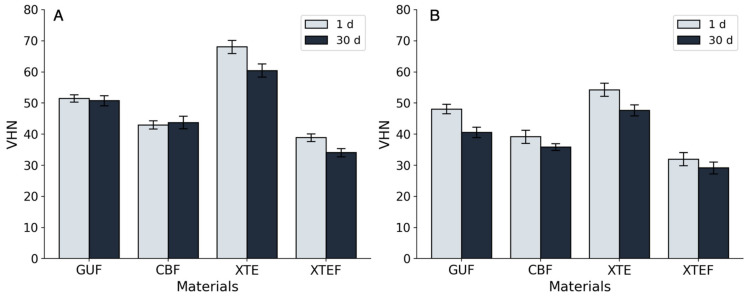
Vickers microhardness (VHN) of the four resin composites after 1 d and 30 d of storage at 37 °C. (**A**) Distilled water; (**B**) 75% ethanol/water. Error bars represent one standard deviation (*n* = 6 per group).

**Figure 4:**
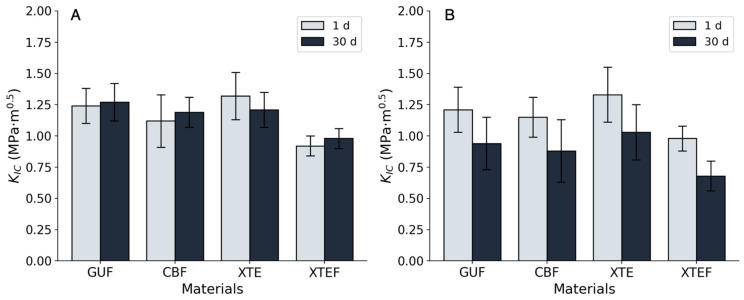
Fracture toughness (K_IC_) of the four resin composites after 1 d and 30 d of storage at 37 °C. (**A**) Distilled water; (**B**) 75% ethanol/water. Error bars represent one standard deviation (*n* = 6 per group).

Descriptive statistics for Vickers microhardness and fracture toughness are given in Table 2 and Table 3, and the corresponding treatment-level comparisons are illustrated in Figure 3 and Figure 4. The three-way ANOVA on VHN identified significant main effects of material (F(3,80) = 858.7, *p* ≤ 0.05), storage medium (F(1,80) = 497.0, *p* ≤ 0.05), and storage time (F(1,80) = 127.0, *p* ≤ 0.05), together with significant material × medium (F(3,80) = 26.5, *p* ≤ 0.05), material × time (F(3,80) = 11.6, *p* ≤ 0.05), medium × time (F(1,80) = 9.0, *p* ≤ 0.05) and three-way material × medium × time (F(3,80) = 8.0, *p* ≤ 0.05) interactions. The corresponding analysis on K_IC_ returned significant main effects of material (F(3,80) = 17.8, *p* ≤ 0.05), medium (F(1,80) = 14.5, *p* ≤ 0.05) and time (F(1,80) = 15.7, *p* ≤ 0.05) and a significant medium × time interaction (F(1,80) = 18.7, *p* ≤ 0.05), whereas the interactions involving material (A × B, A × C, and A × B × C; F ≤ 0.5) did not reach significance, indicating that the four composites responded similarly to the combined effect of solvent exposure and time. Under every condition, the packable nanohybrid composite XTE showed the highest mean VHN and K_IC_, and the conventional flowable XTEF showed the lowest; the two new-generation flowables GUF and CBF occupied an intermediate position. Ethanol/water storage produced larger reductions in both properties than distilled water storage, with the effect being most pronounced after 30 days and most clearly expressed in VHN.

### 3.1. Vickers Microhardness

At 1 d, Filtek Z350 XT (XTE) exhibited the highest mean VHN in both media (distilled water: 68.1 ± 2.1; 75% ethanol/water: 54.3 ± 2.1). G-ænial Universal Flo (GUF) and Charisma Bulk Flow ONE (CBF) showed intermediate values (51.5 ± 1.2 and 43.0 ± 1.3 respectively in water) that were significantly higher than the conventional flowable XTEF (38.3 ± 1.2) but lower than XTE (Tukey, *p* ≤ 0.05) (Figure 3A).

After 30 d of aging, every material showed a decrease in VHN. Softening was medium-dependent: distilled water induced a 5–11% reduction relative to the 1 d water value, whereas 75% ethanol/water induced a 17–30% reduction relative to the same reference (Figure 3B). The hierarchy XTE > GUF > CBF > XTEF was preserved at every sampling point (*p* ≤ 0.05). The largest absolute loss was recorded for XTE (−20.4 VHN units from 1 d water to 30 d ethanol/water), while XTEF showed the largest relative drop (−23.8%).

### 3.2. Fracture Toughness (KIC)

The K_IC_ ranking paralleled the hardness ranking. At 1 d, XTE, GUF and CBF formed a single statistical group with mean toughness of 1.32 ± 0.19, 1.24 ± 0.14 and 1.12 ± 0.21 MPa·m^0.5^ respectively, all significantly higher than XTEF (0.92 ± 0.08 MPa·m^0.5^; Tukey, *p* ≤ 0.05) (Figure 4A). Although XTE showed the highest mean value, the difference relative to GUF and CBF did not reach significance at this time point.

Thirty-day storage in distilled water did not alter K_IC_ in any composite (*p* > 0.05; Figure 4A). Thirty-day storage in 75% ethanol/water, in contrast, reduced K_IC_ in every group by 21–26% relative to the 1 d water value (Figure 4B). The reductions were 24% for GUF (1.24→0.94), 21% for CBF (1.12→0.88), 22% for XTE (1.32→1.03), and 26% for XTEF (0.92→0.68). After 30 d of ethanol/water exposure the four materials separated into four distinct statistical groups, with XTE showing the highest residual K_IC_ (1.03 ± 0.22 MPa·m^0.5^) and XTEF the lowest (0.68 ± 0.12 MPa·m^0.5^; *p* ≤ 0.05). The non-significant material × medium and material × time interactions indicate that the four composites lost a statistically indistinguishable fraction of their initial K_IC_, but the absolute hierarchy was preserved across all conditions.

## 4. Discussion

The present study examined how solvent aging modifies the surface hardness and fracture toughness of four resin composites, representing the contemporary spectrum from conventional flowables to high-strength flowables to a highly filled packable. Three observations stand out. First, the nanohybrid packable XTE showed the highest VHN and K_IC_ under every condition, while the conventional flowable XTEF showed the lowest, with the two high-strength flowables GUF and CBF occupying a clearly intermediate position. Second, 30-day immersion in 75% ethanol/water produced substantially greater reductions in both properties than 30-day immersion in distilled water. Third, water storage for 30 days did not measurably alter K_IC_ for any material, whereas ethanol storage reduced K_IC_ by a comparable percentage (21–26%) in all four composites, indicating that the matrix-dominated fracture mechanism is similarly vulnerable across these formulations. Both null hypotheses are therefore rejected.

The greater impact of ethanol/water relative to water is consistent with the well-documented pattern of solvent-induced degradation in dental polymers [1,17,19]. Ethanol is a more effective swelling agent for dimethacrylate networks than water because its solubility parameter is closer to that of the matrix; it penetrates the crosslinked network more rapidly, relaxes internal stresses at the filler–matrix interphase, and facilitates leaching of unreacted monomer and low-molecular-weight degradation products [17,18]. Ferracane and Berge reported that aging experimental composites in 75% ethanol for 6 months produced KIC reductions of 30–56% with concurrent microhardness loss, the largest losses occurring in materials of lower filler loading or degree of conversion [19]; our 30-day exposure produced reductions toward the lower end of that range in all four materials, consistent with the shorter aging interval and the relatively high filler loading of three of the tested materials. Comparably, the review by Wlodarczyk et al. similarly identified ethanol-based protocols as the most challenging short-term aging condition for methacrylate composites [3].

The milder water-induced response agrees with prior reports that short-term aqueous storage produces only modest changes in K_IC_ [1,22,28]. Water sorption is nonetheless mechanistically active. It hydrolyzes ester linkages in the dimethacrylate backbone, diffuses along the filler–matrix interface, and—in radiopaque barium- or strontium-containing glasses—can attack the silica network through ion leaching, producing siloxane bond cleavage and a self-propagating surface degradation [17,18,29]. Early-stage water uptake may also exert a small plasticizing effect that blunts crack-tip stress concentrations and partially offsets degradation [17], likely contributing to the stability of K_IC_ observed here after 30 d in water. Over clinically relevant timescales, however, this transient toughening is displaced by the progressive loss of interfacial integrity and a measurable drop in VHN and K_IC_ [18,28,30,31,32].

Differences in composition explain the consistent mechanical hierarchy observed across conditions. XTE is a densely packed nanohybrid (~63 vol.% filler) with silica/zirconia nanoclusters and a resin matrix combining Bis-GMA, Bis-EMA, UDMA and TEGDMA. The combination of a dense, well-coupled filler network and a matrix with a lower Bis-GMA fraction is consistent with higher baseline mechanical performance and with better resistance to solvent uptake, because Bis-EMA and UDMA are less hydrophilic than Bis-GMA [17,18,32,33]. The superior VHN of XTE is in line with published values for nanohybrid packable composites [11,12], and its K_IC_ (≈1.3 MPa·m^0.5^) falls within the range typically reported for highly filled hybrid composites [1,13]. GUF and CBF narrow but do not close the gap with XTE: both carry filler loadings around 50 vol.%, an increment over XTEF (~42 vol.%) that is clearly reflected in their higher 1-day VHN and K_IC_. The relative ranking GUF > CBF for VHN may be related to the smaller, more densely packed nanofiller reported for G-ænial Universal Flo [2], while the bulk-fill formulation of CBF trades some filler density for depth-of-cure performance.

Viewed together, the VHN and K_IC_ data suggest that the new-generation flowables have succeeded in narrowing the mechanical gap at baseline without eliminating the filler-driven ceiling on fracture resistance. The response to ethanol aging is informative in a different way: the relative K_IC_ loss is essentially the same for all four materials (21–26%) and the material-level ANOVA interactions did not reach significance, implying that under aggressive solvent exposure the matrix phase and the filler–matrix interphase govern crack resistance more strongly than total filler content. This cross-material equivalence of relative solvent susceptibility, despite an almost three-fold range in baseline performance, is a non-obvious outcome that is not predictable from filler content alone and constitutes the principal mechanistic contribution of this work. This observation is consistent with the view of Lohbauer et al. that matrix plasticization and silane-layer hydrolysis, rather than bulk filler loading, are the rate-controlling steps in the mechanical fatigue and slow crack growth of dimethacrylate composites [34]. It points toward resin chemistry—monomer selection, degree of conversion, and silane coupling stability—as the most promising target for further improvement [35], and also reinforces the long-standing argument that flexural and fracture data on unaged specimens can overstate the clinical durability of a composite and should be complemented by solvent-aged measurements when the material is intended for stress-bearing posterior use [17,18,36].

Several limitations should be noted. The 30-day observation window is short relative to the expected service life of a posterior restoration, and the accelerated-aging medium, although standard, does not reproduce the full chemical and mechanical complexity of the oral environment, including thermal and masticatory cycling, enzymatic attack by salivary esterases [30], and biofilm-driven acidification. In particular, no thermal cycling was applied; thermo-mechanical cycling is a common simulation of the oral environment and should be incorporated in future work to better reproduce in-service thermal and masticatory stresses. The SENB configuration, while consistent with ASTM D5045 and ISO 13586, does not capture the full loading spectrum of a clinical restoration. Finally, no direct measurement of degree of conversion was made; differences in cure between materials cannot be completely excluded and could partially modulate the aging response [32,37]. Post-test fracture surfaces were not examined by scanning electron microscopy; SEM fractography of solvent-aged specimens is recommended in future work to directly visualize the filler–matrix degradation inferred here, as documented for resin composites [38,39].

## 5. Conclusions

Within the limitations of this in vitro study, the packable nanohybrid composite XTE showed the highest Vickers microhardness and fracture toughness under all conditions, whereas the conventional flowable XTEF showed the lowest; the new-generation flowable composites GUF and CBF occupied an intermediate position, narrowing but not eliminating the gap with the packable benchmark. Thirty-day storage in 75% ethanol/water produced a significantly greater reduction in both hardness and fracture toughness than 30-day storage in distilled water, which did not measurably alter KIC. Thus, although the high-strength flowables represent a clear mechanical advance over conventional flowables, their resistance to solvent-induced degradation remains inferior to that of the highly filled packable composite.

## Figures and Tables

**Table 1:** Resin composites evaluated in the study.

Material Code	Composite Name (Type)	Manufacturer	Filler Content (Wt.%)	Shade
GUF	G-ænial™ Universal Flo (nano-hybrid “universal” flowable)	GC Corp., Japan	69% (≈50% vol)	A2
CBF	Charisma^®^ Bulk Flow ONE (bulk-fill flowable)	Kulzer GmbH, Germany	70% (≈46% vol)	(One-shade)
XTE	Filtek™ Z350 XT (nano-hybrid packable)	3M ESPE, USA	78% (≈63% vol)	A2
XTEF	Filtek™ Z350 XT Flowable (nano-hybrid flowable)	3M ESPE, USA	65% (≈42% vol)	A2

**Table 2:** Vickers hardness VHN (standard deviation) of resin composites after 1 d, 30 d storage in two solvents at 37 °C.

Materials	Distilled Water		75% Ethanol/Water	
	1 d	30 d	1 d	30 d
GUF	51.5 (1.2) ^a,1^	50.8 (1.6) ^a,1^	48.1 (1.5) ^a,1^	40.6 (1.7) ^b,1^
CBF	43.0 (1.3) ^a,2^	43.8 (2.0) ^a,2^	39.2 (2.1) ^a,2^	35.9 (1.1) ^b,2^
XTE	68.1 (2.1) ^a,3^	60.5 (2.1) ^b,3^	54.3 (2.1) ^b,3^	47.7 (1.8) ^c,3^
XTEF	38.9 (1.2) ^a,2^	34.1 (1.3) ^b,4^	32.0 (2.1) ^b,4^	29.2 (1.9) ^b,4^

For each solvent, identical letter superscripts indicate no significant difference between different time points (*p* > 0.05). Each solvent is analyzed independently. At each time point, identical number superscripts indicate no significant difference between materials (*p* > 0.05).

**Table 3:** Fracture toughness (standard deviation) of resin composites after 1 d, 30 d storage in two solvents at 37 °C.

Materials	Fracture Toughness K_IC_ (M·Pa·m^0.5^)			
	Distilled Water		75% Ethanol/Water	
	1 d	30 d	1 d	30 d
GUF	1.24 (0.14) ^a,1^	1.27 (0.15) ^a,1^	1.21 (0.18) ^a,1^	0.94 (0.21) ^b,1^
CBF	1.12 (0.21) ^a,1^	1.19 (0.12) ^a,1^	1.15 (0.16) ^a,1^	0.88 (0.25) ^b,2^
XTE	1.32 (0.19) ^a,1^	1.21 (0.14) ^a,1^	1.33 (0.22) ^a,1^	1.03 (0.22) ^b,3^
XTEF	0.92 (0.08) ^a,2^	0.98 (0.08) ^a,2^	0.98 (0.1) ^a,2^	0.68 (0.12) ^b,4^

For each solvent, identical letter superscripts indicate no significant difference over time (*p* > 0.05). At each time point, identical number superscripts indicate no significant difference between materials (*p* > 0.05).

## Data Availability

Data presented in this study are available on request from the corresponding authors.
